# Safety and Efficacy of Stereotactic Body Radiotherapy for Centrally Located Early-Stage Non-small Cell Lung Cancer

**DOI:** 10.7759/cureus.108408

**Published:** 2026-05-07

**Authors:** Mirudhula Chitra Ramesh, Mahadev Potharaju, T Raja, Md Zehran

**Affiliations:** 1 Radiation Oncology, Apollo Specialty Hospitals, Chennai, IND; 2 Medical Oncology, Apollo Specialty Hospitals, Chennai, IND

**Keywords:** central lung tumors, hypofractionation, local control, non-small cell lung cancer, stereotactic body radiotherapy, toxicity

## Abstract

Background: Stereotactic body radiotherapy (SBRT) is widely practiced in the management of peripheral early-stage non-small cell lung cancer (NSCLC); however, its utility for central tumors is controversial due to the risk of damaging major mediastinal organs. This study evaluates safety and tumor control for centrally located early NSCLC using a moderately hypofractionated SBRT protocol.

Methods: Between January 2019 and December 2023, 41 patients with central NSCLC who were deemed unfit or declined surgery (T1-T2a N0 M0, tumor diameter ≤ 4 cm) were treated with SBRT (8 Gy × 8 fractions; total dose 64 Gy, BED₁₀ = 115.2 Gy). Central tumors were defined as lesions within 2 cm of the proximal bronchial tree or abutting mediastinal structures. The key outcome was freedom from local progression (FFLP) at three years. Secondary outcomes were regional recurrence-free survival (RRFS), distant recurrence-free survival (DRFS), overall survival (OS), and treatment-related adverse events.

Results: The median age was 69.0 years (range: 40-89 years); 65.9% were male. Median tumor diameter was 2.5 cm, and 65.9% measured 2-4 cm. The three-year FFLP was 85.9% (95% CI: 66.1%-94.5%). Three-year OS, RRFS, and DRFS were 68.6% (95% CI: 48.7%-82.1%), 76.8% (95% CI: 56.6%-88.5%), and 83.6% (95% CI: 66.8%-92.3%), respectively. Local failure occurred in four (9.8%), regional failure in nine (22.0%), and distant failure in eight (19.5%) patients. Treatment-related toxicity was minimal (grade ≥ 2 occurrences in 1 patient (2.4%) and no grade ≥ 3 toxicity).

Conclusions: A dose of 64 Gy delivered in eight fractions through SBRT exerts good local control and low toxicity for centrally located early-stage NSCLC. This hypofractionated regimen seems to be safe and effective for patients who refuse or are unsuitable for surgery and comparable to results achieved with surgery.

## Introduction

Lung cancer is the leading cause of cancer-related mortality worldwide, with non-small cell lung cancer (NSCLC) accounting for approximately 85% of cases [1]. Early-stage NSCLC is primarily managed with surgery, achieving five-year survival rates above 70% in patients with stage I disease [2]. However, a large proportion of patients with early-stage NSCLC are not surgical candidates due to advanced age, poor cardiopulmonary reserve, or associated comorbid conditions. In addition, some patients decline surgery even when it is feasible [3]. Stereotactic body radiotherapy (SBRT), also known as stereotactic ablative radiotherapy (SABR), has been developed as a non-invasive treatment option for early-stage NSCLC. SBRT delivers highly focused ablative radiation to the tumor while minimizing exposure to adjacent normal tissues through steep dose gradients, image guidance, and motion management [4].

Numerous prospective studies and meta-analyses have demonstrated excellent local control with SBRT (over 90% for peripheral lesions at three years), with low toxicity comparable to surgery in medically inoperable patients [5-7]. The RTOG 0236 trial was pivotal in establishing SBRT for peripheral early-stage NSCLC, reporting a three-year primary tumor control rate of 97.6%, with regimens of 54 Gy in three fractions and 48 Gy in four fractions [8]. Subsequent randomized trials comparing surgery and SBRT, including a pooled analysis of the STARS and ROSEL trials, reported similar overall survival (OS) between the two approaches [9]. These findings were further supported by the SABR-COMET trial, which demonstrated the benefit of curative-intent SBRT in selected metastatic settings and reinforced its role in early-stage disease [10].

Despite its success in peripheral tumors, the use of SBRT for centrally located lung tumors remains controversial. Central lesions, defined as those within 2 cm of the proximal bronchial tree or adjacent to mediastinal structures such as the esophagus, heart, and major vessels, pose increased risk due to their proximity to organs at risk (OARs) [11]. Early reports of serious, potentially fatal toxicities following high-dose SBRT for central lesions raised significant safety concerns [12,13]. As a result, many early trials excluded central tumors and explored lower dose-per-fraction regimens to reduce the risk of severe toxicity. The NRG Oncology/RTOG 0813 phase I/II trial evaluated the safety of SBRT for central tumors using dose escalation from 10 Gy × 5 to 12 Gy × 5 fractions [14]. The study identified 10 Gy × 5 fractions (total dose 50 Gy, BED₁₀ = 100 Gy) as a safe and effective regimen, with acceptable rates of high-grade toxicity and encouraging local control. However, the relatively lower BED compared to peripheral regimens raised concerns about potential compromise in tumor control. To address this, alternative fractionation schedules, including 8-10 fraction regimens, have been explored to deliver higher BED while reducing dose per fraction to protect nearby OARs [15-17].

These moderately hypofractionated regimens may reduce the risk to critical structures while maintaining an ablative tumor dose. However, evidence on their long-term safety and efficacy remains limited, and the optimal balance between tumor control and toxicity for central NSCLC is still unclear. To address these issues, this study evaluated whether an SBRT regimen of 8 Gy × 8 fractions (total dose 64 Gy, BED₁₀ = 115.2 Gy) could achieve adequate tumor control with acceptable safety in patients with centrally located early-stage NSCLC. This regimen was selected to approximate the BED used for peripheral tumors while lowering the dose per fraction to reduce the risk of toxicity to adjacent critical structures. We hypothesized that this moderately hypofractionated schedule would provide effective local control with acceptable toxicity in patients who are medically inoperable or who decline surgery.

## Materials and methods

Study design and patient selection

All patients were treated at a single tertiary cancer center, and data were collected retrospectively from January 2019 to December 2023. Institutional review board approval was not required as per institutional protocol, given the retrospective nature of the study. Written informed consent was obtained from all participants. Eligible patients had histologic or cytologic confirmation of NSCLC, clinical stage T1-T2a N0 M0 according to the AJCC 8th edition, and a maximum tumor diameter of ≤4 cm. Tumors were centrally located, defined as within 2 cm of the proximal bronchial tree (trachea, carina, main bronchi, lobar bronchi, bronchus intermedius, and segmental bronchi) or adjacent to the mediastinal pleura or other critical mediastinal structures. Patients were considered medically inoperable due to poor cardiopulmonary reserve, significant comorbidities, or refusal of surgery despite being operable candidates. Additional inclusion criteria included ECOG performance status 0-2, adequate pulmonary function (FEV₁ ≥ 30% predicted or DLCO ≥ 30% predicted), and age ≥18 years. Among the 41 patients included, 39 had adenocarcinoma and 2 had squamous cell carcinoma. Three patients had ultracentral tumors. Exclusion criteria included prior thoracic radiotherapy, evidence of nodal or distant metastases, another active malignancy within the past three years (except non-melanoma skin cancer or carcinoma in situ), pregnancy or lactation, and inability to tolerate the treatment position or comply with follow-up. Staging investigations included contrast-enhanced computed tomography (CT) of the chest and upper abdomen, fluorodeoxyglucose positron emission tomography-CT (FDG PET-CT), and brain magnetic resonance imaging (MRI) or contrast-enhanced CT when indicated. Endobronchial ultrasound (EBUS) was not performed for mediastinal staging. Pulmonary function tests and cardiopulmonary evaluation were used to determine surgical inoperability or to document patient refusal. All cases were reviewed in a multidisciplinary tumor board, which confirmed SBRT as an appropriate treatment option.

Treatment planning

All patients underwent CT simulation in the treatment position using appropriate immobilization (vacuum cushion with arms raised above the head). Respiratory motion and tumor mobility were assessed using four-dimensional CT (4D-CT). Imaging was performed with 1 mm slice thickness from the mandible to the upper abdomen, and intravenous contrast was administered unless contraindicated. In accordance with institutional protocols and published guidelines, experienced radiation oncologists contoured target volumes and OARs [18,19]. The gross tumor volume (GTV) was defined on lung window CT images with input from PET-CT. The internal target volume (ITV) was generated by combining GTV contours across all respiratory phases from the 4D-CT to account for tumor motion. A uniform 5 mm margin was added to the ITV to create the planning target volume (PTV), accounting for setup uncertainties and residual motion. OARs were contoured using established atlases and included the proximal bronchial tree, esophagus, heart, great vessels (aorta and pulmonary artery), spinal cord, lungs (total lung minus GTV), chest wall, trachea, and brachial plexus. Dose constraints were applied according to RTOG 0813 and AAPM TG-101 recommendations for central lung lesions [14,20]. Key constraints included proximal bronchial tree D₀.₀₃cc <64 Gy, esophagus D₀.₀₃cc <35.2 Gy, heart D₀.₀₃cc <48 Gy, great vessels D₀.₀₃cc <56 Gy, spinal cord D₀.₀₃cc <24 Gy, and mean lung dose <12 Gy. All patients were treated using the CyberKnife VSI system with the Multiplan treatment planning system (Accuray, Sunnyvale, CA, USA). Fiducial-free Synchrony respiratory tracking was used for continuous, real-time tumor tracking and image guidance. Treatment was delivered five days per week, with weekends as rest days.

Follow-up and outcome assessment

Patients were evaluated at baseline, weekly during treatment, and at regular intervals after SBRT. Post-treatment follow-up was scheduled at 3 months, 6 months, 12 months, and every 6 months thereafter. Each visit included a clinical examination and contrast-enhanced CT of the chest. PET-CT was performed when clinically indicated to evaluate suspicious findings or confirm recurrence. Toxicities were recorded at each visit and graded according to CTCAE version 5.0 [21]. Acute toxicity was defined as events occurring within 90 days of SBRT completion, while late toxicity was defined as events occurring beyond 90 days. Recurrences were classified as local, regional, or distant. Local recurrence was defined as disease progression within or directly adjacent to the PTV. Regional recurrence included involvement of hilar or mediastinal lymph nodes. Distant recurrence was defined as disease occurring beyond the ipsilateral hemithorax. Recurrence was confirmed by biopsy whenever feasible or by radiographic progression on serial imaging, with or without metabolic progression on PET-CT. The primary endpoint was three-year freedom from local progression (FFLP), measured from the start of SBRT to the time of local recurrence or last follow-up. Secondary endpoints included regional recurrence-free survival (RRFS), distant recurrence-free survival (DRFS), and overall survival (OS), defined as the time from SBRT initiation to the respective event or last follow-up.

Statistical analysis

Descriptive statistics were used to summarize patient, tumor, and treatment characteristics. Continuous variables were reported as mean, median, standard deviation, and range. Categorical variables were presented as counts and percentages. Survival outcomes were estimated using the Kaplan-Meier method, with 95% CIs calculated using the Greenwood method. Survival time was measured from the start of SBRT to the event of interest or the last follow-up for censored patients. Patients without an event were censored at the time of last contact. All analyses were performed using R (R Foundation for Statistical Computing, Vienna, Austria), utilizing the survival and survminer packages. Statistical significance was defined as a two-sided p-value < 0.05.

## Results

Patient and tumor characteristics

A total of 41 patients with centrally located early-stage NSCLC underwent SBRT as per protocol between January 2019 and December 2023. At the time of treatment, the median age was 69.0 years (mean 67.2 ± 9.6 years; range 40-89). Twenty-seven patients (65.9%) were male, and 14 (34.1%) were female. All patients had an ECOG performance status of 0-2, with most having a status of 0 or 1. Fourteen patients (34.1%) had lesions measuring ≤2 cm, while 27 patients (65.9%) had tumors measuring 2.1-4 cm (Table 1). All tumors met the protocol definition of central location, being within proximity to the proximal bronchial tree, mediastinal pleura, or other critical mediastinal structures. The majority of patients were medically inoperable due to cardiopulmonary limitations or comorbidities, while a small proportion declined surgery despite being operable candidates.

**Table 1:** Patient and tumor characteristics SBRT: stereotactic body radiotherapy, FU: follow-up.

Characteristic	Overall	≤2 cm (n = 14)	>2 cm (n = 27)	Test statistic	p-value
Median age (range)	69.0 (40-89)	69	69	U = 198.0	0.815
Male, n (%)	27 (65.9)	11 (78.6)	16 (59.3)	Chi-square = 1.529	0.216
Female, n (%)	14 (34.1)	3 (21.4)	11 (40.7)		
Median FU, months (range)	34.0 (24-46)	36	32	U = 267.0	0.032

All 41 patients completed the prescribed SBRT dose of 64 Gy in eight fractions without treatment interruptions or dose reductions. The median treatment duration was 10 days. All treatment plans complied with OAR constraints as per RTOG 0813 and AAPM TG-101 guidelines. Plan quality assurance was acceptable for all patients, with measured and calculated doses within institutional tolerance limits. No treatment-related complications requiring hospitalization occurred during therapy.

**Figure 1:**
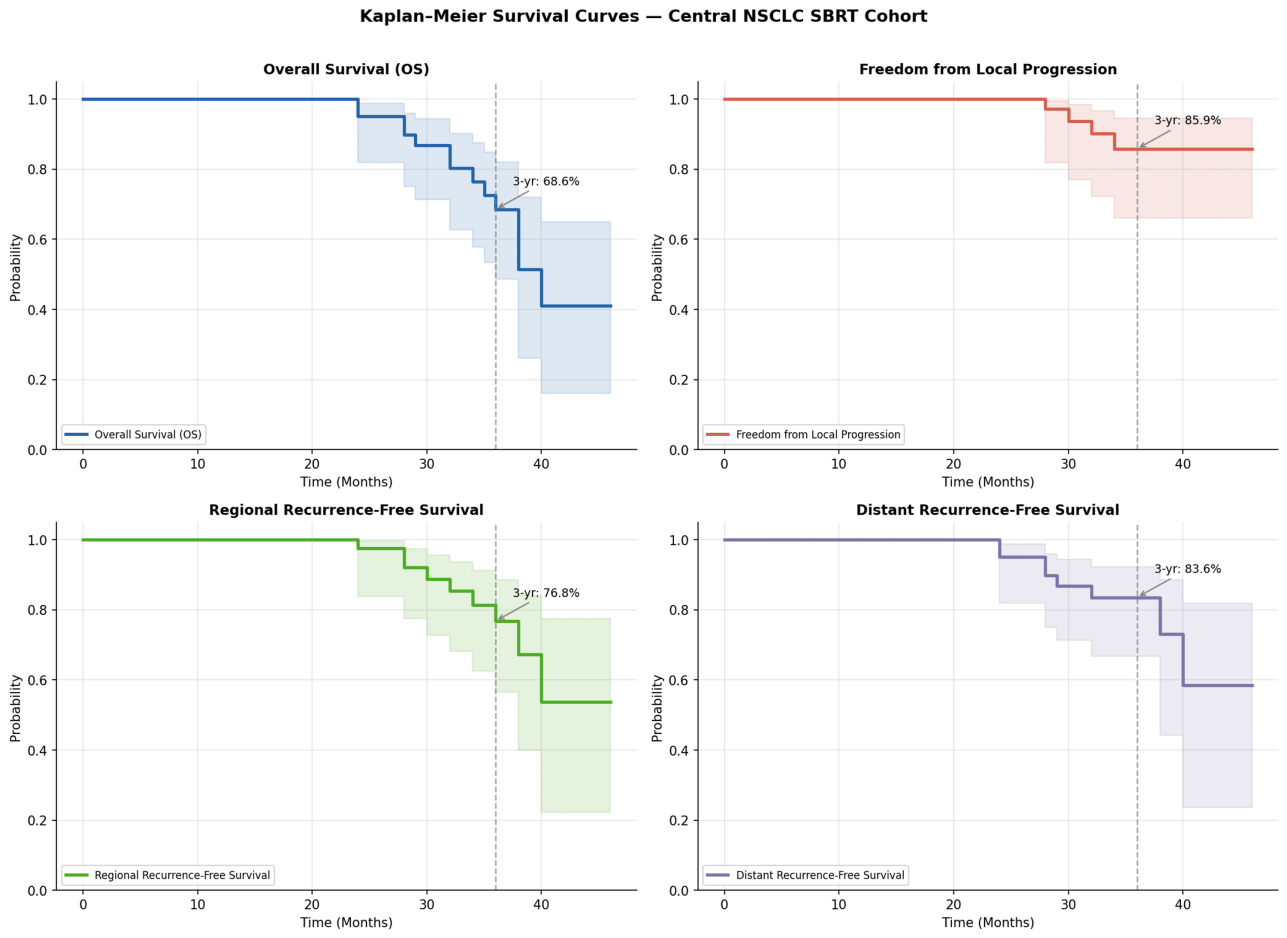
Kaplan-Meier survival curves All endpoints demonstrate favorable outcomes at three years of follow-up. Shaded areas represent 95% confidence intervals; dashed vertical lines indicate 36 months.

The median follow-up was 34.0 months (mean 33.2 ± 5.4 months; range 24-46). Thirteen patients (31.7%) died during follow-up. Local recurrence occurred in four patients (9.8%), regional recurrence in nine patients (22.0%), and distant metastases in eight patients (19.5%). The three-year FFLP was 85.9% (95% CI: 66.1%-94.5%). Kaplan-Meier analysis (Figure 1) showed FFLP rates of 100.0% at 12 and 24 months (95% CI: 100.0%-100.0%), decreasing to 85.9% at 36 months. All four local recurrences occurred between 24 and 36 months after SBRT initiation. The three-year OS was 68.6% (95% CI: 48.7%-82.1%). The Kaplan-Meier OS rates were 100.0% at 12 months (95% CI: 100.0%-100.0%), 95.1% at 24 months (95% CI: 81.9%-98.8%), and 68.6% at 36 months. Most deaths occurred during the third year of follow-up (Table 2).

**Table 2:** Kaplan-Meier survival estimates for the overall cohort and by tumor size group Between-group comparisons (tumor size ≤2 cm vs. >2 cm) were performed using the log-rank test; the test statistic is reported as the log-rank χ^2^ value. OS: overall survival, FFLP: freedom from local progression, RRFS: regional recurrence-free survival, DRFS: distant recurrence-free survival.

Endpoint	12 months, % (95% CI)	24 months, % (95% CI)	36 months, % (95% CI)	3 years (≤2 cm) (%)	3 years (>2 cm) (%)	Log-rank, χ^2^	p-value
OS	100.0	95.1 (81.9-98.8)	68.6 (48.7-82.1)	91.7	53.8	4.724	0.030
FFLP	100.0	100	85.9 (66.1-94.5)	100	77.0	2.635	0.105
RRFS	100.0	97.6 (83.9-99.7)	76.8 (56.6-88.5)	100	62.9	6.829	0.009
DRFS	100.0	95.1 (81.9-98.8)	83.6 (66.8-92.3)	91.7	79.3	2.398	0.121

In total, 17 patients (41.5%) experienced some form of recurrence. Local recurrence at the primary site was observed in four patients (9.8%), all occurring between 24 and 36 months after treatment. Regional recurrence occurred in nine patients (22.0%), often presenting as isolated nodal failure without concurrent local or distant disease. Distant metastases were seen in eight patients (19.5%), with common sites including the contralateral lung, bone, and brain. Some patients developed multiple sites of recurrence either simultaneously or over time. The relatively low rate of local recurrence (9.8%) suggests effective local tumor control with this SBRT regimen. However, the higher rates of regional and distant failure highlight the systemic nature of lung cancer and the need for careful surveillance and consideration of systemic therapy in selected patients.

Treatment-related adverse events were generally mild and well tolerated. Most acute toxicities, occurring during or within 90 days of SBRT, were grade 1 and included fatigue, cough, and mild dyspnea. Only one patient (2.4%) experienced grade 2 acute toxicity (radiation pneumonitis), which resolved with a short course of corticosteroids. No grade 3 or higher acute toxicities were observed. Late toxicities (>90 days) were also uncommon, with no grade 3 or higher events reported during follow-up. There were no cases of bronchial stenosis, esophageal stricture, clinically significant pericardial effusion, or other serious complications. No treatment-related deaths occurred. These favorable toxicity outcomes support the safety of the 8 Gy × 8 fraction SBRT regimen for central lung tumors when careful planning, strict adherence to OAR constraints, and precise image-guided delivery are employed.

## Discussion

This retrospective single-institution cohort analysis suggests SBRT with 64 Gy in eight fractions obtains good local control, with minimal toxicity in early-stage, centrally located NSCLC patients. The reported three-year FFLP of 85.9% and grade 3 (or later) toxicity absence indicate safety and efficacy for this moderate hypofractionated therapy for central lesions in medically inoperable or surgery-unwilling patients. SBRT has changed the landscape of centrally located early-stage NSCLC treatment in the last two decades. Experiments with very large per-fraction doses (e.g., 20 Gy × 3 or 18 Gy × 3) for central tumors, in the first phases of treatment, produced unacceptable rates of severe complications, including severe hemoptysis, bronchial necrosis, and esophageal trauma [12,13]. These results ultimately encouraged the selection of revised fractionation schedules to reduce catastrophic toxicity while maintaining tumor control.

The NRG/RTOG 0813 trial characterized the 10 Gy × 5 fractions (50 Gy total, BED₁₀ = 100 Gy) as a suitable and effective regimen for central tumors based on two-year primary tumor control of 87.9% and manageable grade ≥3 toxicity [14]. However, due to RTOG 0813 providing less BED compared to most peripheral regimens, long-term local control is still a concern. Decreasing local control has been reported for lower BED schedules even after three years by other institutions [22]. Our 8 Gy × 8 fractions regimen (64 Gy total, BED₁₀ = 115.2 Gy) results in a higher BED than RTOG 0813 and a smaller per fraction dose relative to ultra hypofractionated routines. From a radiobiological standpoint, decreasing dose per fraction minimizes late toxicity risk to serial organs (e.g., bronchial tree and esophagus), but still ensures ablative total BED for tumor suppression [23].

Our three-year FFLP of 85.9% is similar to results reported for central tumor SBRT at different fractionations [14,15,24]. Various institutions have described beneficial results with a moderately hypofractionated approach: Haasbeek et al. reported 63 central tumors managed with risk-adapted SBRT (8 Gy × 7 fractions), at or near-critical structures, and reported three-year local control of 93% with acceptable toxicity [15]. Rowe et al. reported three-year local control of 87.5% with 7.5 Gy × 8 fractions, and no grade 4-5 toxicity [25]. These results correlate with our results and recommend moderately hypofractionated strategies to maximize the therapeutic ratio for central NSCLC. The toxicity profile in our cohort was very favorable, because only one patient (2.4%) had grade 2 toxic effects, and there were no grade ≥3 events. In contrast, RTOG 0813 found grade ≥3 toxicity of 7.2% of treated patients with fractions of 10 Gy × 5 [14]. We reasoned that the eight fractions used (lowering per-fraction dose to OARs), strict adherence to OAR restrictions, continuous tumor tracking, appropriate patient selection, and multidisciplinary review were key contributors to the low toxicity rates observed here. There has been an association of particularly high toxicity of these "ultra-central" tumors, lesions surrounding or overlapping the proximal bronchial tree or esophagus [26]. While we did not conduct a separate analysis on ultra-central lesions, the overall low toxicity indicates that the eight-fraction regimen still offers a safety net even in lesions proximal to central critical structures. We obtained an OS of three years of 68.6% in our series, consistent with published SBRT results in early-stage NSCLC in medically inoperable groups [8,14,27]. OS in these populations is dominated by competing mortality associated with comorbidities and advancing age. Comorbidity burden was substantial, our median cohort age was 69 years, and all patients were either medically inoperable or refused surgery. Cancer-specific survival would have probably exceeded OS, but no competing risk-based analyses were performed. Patterns of failure in this study are instructive. The low local recurrence rate (9.8%) indicates successful local tumor eradication with SBRT intervention. In contrast, recurrences from both regional (22.0%) and distant (19.5%) locations were more common, a striking phenomenon consistent with systemic NSCLC patterns at this early stage.

These data suggest that although SBRT is an excellent local treatment option, in certain patients, occult nodal or distant disease may be present at the outset and become apparent through follow-up. The comparatively high rate of regional recurrence deserves attention, too. Many series demonstrate failure rates of 5%-15% regionally post-SBRT for early-stage NSCLC [8,28,29], with some reports indicating higher rates for central lesions [30]. Possible factors to consider include occult nodal involvement not noticed at staging time, lymphatic drainage patterns in central tumors, avoidance of elective nodal radiation in SBRT, and lack of invasive mediastinal staging for medically inoperable patients. Of the patients, 19.5% developed distant metastases, which is consistent with the 10%-20% reported for early-stage disease in SBRT patients [8]. The most frequent metastatic sites were the contralateral lung, bone, and brain. These results underscore the need for extensive surveillance imaging and systemic therapy, particularly in high-risk patients.

This study has several limitations. This is a single-institution study with a small sample size (41 patients), limiting the power to detect differences in other outcome groups. These findings warrant the validation of larger multi-institutional studies and prognostic factors. Our median follow-up at 34 months, appropriate for the three-year endpoints, is short and cannot assess long-term local controls and late toxicity, hence requiring longer follow-up. The cohort was heterogeneous regarding reasons for inoperability, tumor size, and histology, which might have affected results. Dose and toxicity dosimetric correlations for some OAR doses were not conducted and would benefit from more toxicity events in a larger dataset. Another important limitation is the absence of routine EBUS as a staging procedure in our center. This procedure could have picked up occult nodal involvement. Finally, quality-of-life measurements are not systematically collected, but patient-reported outcomes are becoming more important for the assessment of treatment. Notwithstanding these caveats, this series adds further evidence regarding the safety and efficacy of moderately hypo-fractionated SBRT for centrally located early-stage NSCLC. The eight-fraction design is practical in most of the centers using modern linacs and planning systems, and its favorable toxicity and good local control are conducive to its application as a treatment modality in inoperable patients with central tumors. Future works should involve more extended follow-up to evaluate the durability of control effects and late consequences, trials comparing fractionation schedules for central tumors and identification of molecular biomarkers, and the use of circulating tumor cells to identify patients at high risk for regional and distant relapse who might benefit from systemic intervention.

## Conclusions

SBRT delivering 64 Gy in eight fractions achieves excellent local control, acceptable survival outcomes, and a safety profile for centrally located early-stage NSCLC. This moderately hypofractionated regimen represents a clinically meaningful and radiobiologically rational treatment option for medically inoperable patients or those who decline surgery. Outcomes are competitive with those of more aggressive fractionation schedules while carrying a substantially lower risk of severe toxicity. Tumor size is a significant determinant of regional control and OS and should be taken into consideration in patient counselling and post-treatment surveillance strategies. These results contribute meaningfully to the evolving evidence base for central NSCLC management and provide a strong foundation for future prospective trials aimed at further optimizing outcomes in this challenging patient population.
